# A comparison of airway management devices in simulated entrapment-trauma: a prospective manikin study

**DOI:** 10.1186/s12245-019-0233-z

**Published:** 2019-07-08

**Authors:** Robin Pap, Charl van Loggerenberg

**Affiliations:** 1 School of Science and Health, Western Sydney University, Locked Bag 1797, Penrith, Sydney, NSW 2751 Australia; 2 ER Consulting Inc., Johannesburg, South Africa; 3 School of Clinical Medicine, University of the Witwatersrand, Johannesburg, South Africa

**Keywords:** Airway management, Traffic accidents, Endotracheal intubation, Laryngeal mask airway

## Abstract

**Introduction:**

In the patient entrapped after a motor vehicle collision (MVC), advanced airway management may need to be performed before extrication. The aim of this study was to compare four airway management devices utilized by paramedics in a simulated entrapped patient.

**Methods:**

Twenty-six paramedics performed advanced airway management on a manikin seated in the driver’s seat (right side) of a car. Access was through the opened door only. The airway devices were the Macintosh laryngoscope and the Airtraq optical laryngoscope to facilitate the endotracheal intubation (ETI), the laryngeal mask airway (LMA) Supreme and the laryngeal tube (LT). Time to first successful ventilation and number of attempts required for successful placement were measured. Following each placement, participants rated the degree of difficulty. For ETI, participants ranked the achieved glottic view using Cormack-Lehane grades (CLG). Finally, participants were asked which airway management device they preferred.

**Results:**

The LMA Supreme had the shortest mean time to first successful ventilation (16.7 s, CI [0.95] 14.9–18.6). Insertion of the LMA Supreme and ETI with the Macintosh laryngoscope had 100% first-attempt success. The LMA Supreme was rated least difficult to insert (mean score 1.7/10 (CI [0.95] 1.2–2.1)). Compared to the Macintosh, the Airtraq laryngoscope facilitated superior laryngoscopy (CLG I view 46.2% and 80.8%, respectively). Most participants (10/26; 38%) chose the Macintosh laryngoscope as their preferred technique, followed closely by the LMA Supreme (9/26; 35%).

**Conclusion:**

The LMA Supreme took the least amount of time and was the easiest to be inserted. Extraglottic airway devices may be beneficial alternative airway management devices to be considered by paramedics in the entrapped patient. Endotracheal intubation using the Macintosh laryngoscope was performed competently by participating paramedics. The Airtraq enabled superior laryngoscopy but resulted in poorer first-pass success rate.

## Background

High-speed motor vehicle collisions (MVCs) can produce significant mechanical damage to the cars involved and are likely to entrap the front occupants. The term ‘entrapment-trauma’ describes this patient collective in prehospital emergency care [1]. Severely injured patients frequently require endotracheal intubation (ETI) [2, 3]. However, airway management in entrapment-trauma is challenging due to restricted access, the seated position of the patient and the need for cervical spinal immobilization [4–6]. This may result in harmful delay or even failure to provide oxygenation and ventilation.

Although the value of ETI in the prehospital environment is widely debated [7, 8], the cuffed endotracheal tube is considered the gold standard of airway management devices in any setting. The endotracheal tube is traditionally placed with a laryngoscope using a Macintosh blade (Macintosh laryngoscope). The success rate of prehospital endotracheal intubation varies greatly depending on several factors including patient characteristics, procedural method (rapid sequence intubation versus non-drug-facilitated intubation) and clinician background and experience [6]. The incidence of failed intubation may be especially high in entrapment-trauma [5]. Video or optical laryngoscopy provides an alternative after a failed direct laryngoscopy attempt, but also a method for best first attempt in the anticipated difficult airway. Indirect laryngoscopy devices have been investigated extensively in situations with full access [9–13] as well as restricted access [14–20]. The Airtraq optical laryngoscope (Prodol Meditec, Vizcaya, Spain) may be a cost-effective, single-use device for prehospital ETI of the anticipated difficult airway in the entrapped patient [18, 21].

Extraglottic airway devices are routinely used as rescue devices when endotracheal intubation has failed; however, their utilization as a primary alternative advanced airway management device has been suggested [22, 23]. This may be especially advocated when basic airway techniques fail or when access to the patient inhibits intubation attempts. Facemask ventilation is a basic yet challenging skill especially in the patient with restricted access. A high potential of achieving only inadequate facemask seal and associated leak causing insufficient lung ventilation exists [24]. Additionally, the risk of gastric insufflation is high [25]. The specific choice of alternative airway management device will depend on availability and clinician skill, experience and preference [26]. The laryngeal mask airway (LMA) Supreme (Teleflex, Athlone Co. Westmeath, Ireland) and the laryngeal tube (LT) (VBM Medizintechnik, Sulz, Germany) in entrapment-trauma have been studied [14, 18, 27]; however, clinician preference was not considered.

In this study, time to first successful ventilation, number of attempts required for success insertion, perceived insertion difficulty, Cormack-Lehane grades (CLG) achieved with the laryngoscopes and clinician preference were measured to compare the Macintosh laryngoscope, the Airtraq optical laryngoscope, the LMA Supreme and the LT used by paramedics in the simulated entrapped patient.

## Methods

This was a prospective manikin study. The study was conducted at the Department of Emergency Medical Sciences of the Cape Peninsula University of Technology, Cape Town, South Africa. A convenience sample of 26 advanced life support (ALS) paramedics from the Western Cape Government METRO Emergency Medical Services and private ambulance service organizations in the Western Cape region, South Africa, volunteered to participate in this study. Participants were asked to complete a short questionnaire at the start of four separate data collection days. The questionnaire asked about their paramedic qualifications, whether they had previously practiced with the Laerdal ALS Simulator manikin, length of experience at ALS paramedic level, self-estimated number of situations in which they needed to provide advanced airway management in an entrapped patient per year, and what airway management devices they carried and could use in their clinical practice. On each data collection day, an extensive training workshop was conducted prior to data collection which included theoretical and practical training on the airway management devices and their use including the specific scenario under investigation. The workshops aimed at making participating paramedics competent in using all devices included in the study.

**Fig. 1:**
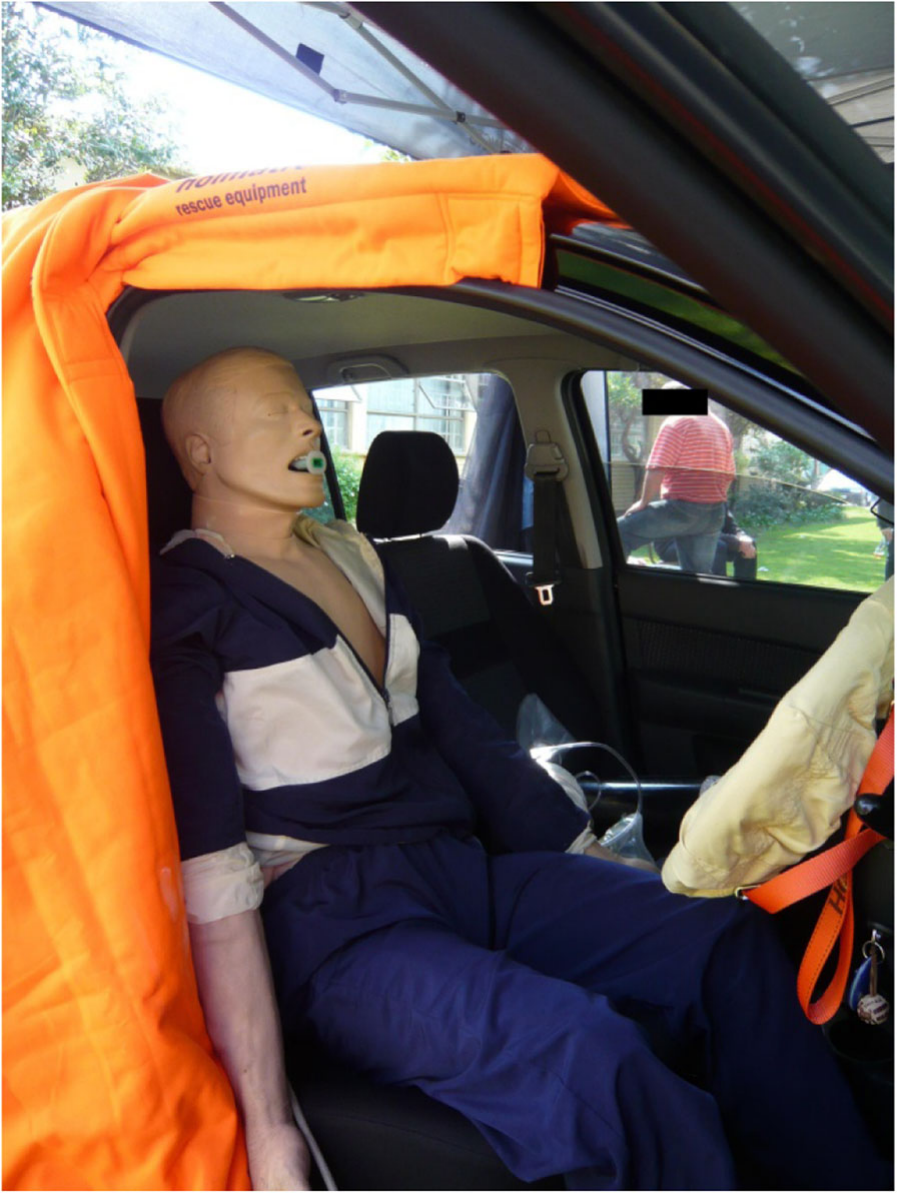
Photograph of the simulated entrapped patient in the driver’s seat of the car with opened door

The entrapped patient was simulated by positioning a manikin (Laerdal ALS Simulator, Laerdal Medical, Stanvenger, Norway) as the entrapped driver in a light motor vehicle (Hyundai Getz, 2008) parked outdoors during daytime (Fig. 1).

The vehicle was positioned under a gazebo to eliminate weather variables. Normal amounts of airway secretions were simulated with Laerdal airway manikin lubricant and kept constant throughout data collection. All airway devices were lubricated before insertion procedures. An assistant maintained consistent cervical spine immobilization from the back seat behind the patient, and the cervical collar was undone to facilitate mouth opening as a standardized practice during the airway interventions. The assistant was not permitted to apply any laryngeal manipulation to potentially improve laryngoscopy or placement. Access was limited to that from the opened driver’s door (right), i.e. face-to-face position. On each of the data collection days, attending paramedics inserted all of the following advanced airway devices in randomized sequence:

- ETI with a Macintosh laryngoscope (KaWe, Asperg, Germany), blade size 3 and endotracheal tube ID 7.5 mm with stylet
- ETI with the Airtraq optical laryngoscope (direct view without additional equipment) (Prodol Meditec, Vizcaya, Spain), size 3 and endotracheal tube ID 7.5 mm
- Laryngeal mask airway Supreme (Teleflex, Athlone Co. Westmeath, Ireland), size 4
- Laryngeal tube suction disposable (VBM Medizintechnik, Sulz, Germany), size 4

Time was measured with a stop watch, from when simulated facemask ventilation was stopped to first ventilation through the endotracheal tube or extraglottic device. An in situ oropharyngeal airway needed to be removed prior to advanced airway management. All participants were also video recorded by a camera mounted inside the vehicle on the rear-view mirror. Successful placement was defined as the airway device in the correct position with cuff/mask inflated. This was assessed by visual inspection of the device inside the manikin’s airway after the timed scenario and recorded as the number of attempts required for successful placement for each device. A maximum of three attempts were allowed per device. Participants did not receive any feedback between attempts. Each attempt was allowed to continue for a maximum of 120 s after which the participant was stopped and the attempt was recorded as unsuccessful. In a second questionnaire, participants were asked to record the CLG [28] they achieved during their endotracheal intubations. Degree of difficulty was measured using a 1 to 10 rating scale for all airway management devices (1 being least difficult and 10 being most difficult). Participants completed the questionnaire immediately after using each device. After having used all devices, the questionnaire also inquired about personal preference.

The primary outcomes were time to first successful ventilation, number of attempts required for success placement and degree of difficulty. Secondary outcomes were CLG achieved with the laryngoscopes and clinician preference.

Quantitative data were analysed using Stata 10.0 statistics/analysis software by StataCorp (Texas, USA). The data analyses for this study were performed in three phases. Firstly, descriptive statistics were computed for all variables of interest. Secondly, Shapiro-Wilk tests were conducted for normality on all relevant variables. This informed statistical test choice for both the univariate and multivariate analysis. Due to the distribution characteristics of the data, non-parametric statistics were used, namely Kruskal-Wallis for the analysis of variance and Wilcoxon’s rank sum (Mann-Whitney *U*) and chi-square tests (nominal variables) to conclude testing for statistical significance between individual variables and pooled data. The level of statistical significance (alpha) used was .05. Thirdly, relationships between variables were tested using Spearman’s rho and multiple regression analysis. Multiple regression was performed using automatic forward stepwise models to examine relationships between three or more variables while Spearman’s rho was used when testing relationships between only two variables.

The Human Research Ethics Committee of the University of the Witwatersrand, Johannesburg, South Africa, granted approval for this research. All participants were informed in writing and verbally about details of the workshop, data collection and the study overall. Participation was voluntary and allowed to be discontinued at any time. All participants signed an informed consent form.

## Results

All 26 participating paramedics had completed a university qualification in paramedicine with a minimum full-time duration of 3 years. All participants had previously practiced with the Laerdal ALS Simulator manikin during their paramedic training or thereafter. The mean operational paramedic experience of the participants at the time of data collection was 38.8 months (CI [0.95] 22.1–55.5). The mean number of times participants estimated they apply airway management in entrapment-trauma per year was 4.3 (CI [0.95] 2.8–5.8). All participants indicated that they were equipped with a Macintosh laryngoscope when performing their clinical duties. One participant (3.8%) had the LT, and 22 (84.6%) carried other extraglottic airway devices in their kits, namely standard LMAs (*n* = 18; 69.2%) or the iGel (Intersurgical, Berkshire, UK) (*n* = 4; 15.4%). Three participants (11.5%) carried no extraglottic airway devices, and no one participating in the study had access to video or optical laryngoscopy equipment in their clinical practice.

### Time to first successful ventilation

The mean time to first successful ventilation was shorter with the LMA Supreme (16.7 s, CI [0.95] 14.9–18.6) and the LT (19.4 s, CI [0.95] 18.0–20.8) compared to endotracheal intubation using the Macintosh laryngoscope (37.7 s, CI [0.95] 31.8–43.5) and the Airtraq (41.2 s, CI [0.95] 36.7–45.6) (*p* < 0.001; Table 1).

**Table 1:** Time to first successful ventilation (sec)

	Mean	Median	SD	95% CI
LMA Supreme	16.7	16.0	4.5	14.9–18.6
Laryngeal tube	19.4	19.0	3.4	18.0–20.8
Macintosh	37.7	33.5	14.4	31.8–43.5
Airtraq	41.2	39.5	11.0	36.7–45.6

*SD* standard deviation, *CI* confidence interval, *LMA* laryngeal mask airway

Extraglottic airway devices had a significantly shorter time to first successful ventilation compared to the laryngoscopes (*p* < 0.001). The Macintosh and Airtraq laryngoscopes were not significantly different in terms of time to first successful ventilation (*p* = 0.17); however, the observed difference between the two extraglottic airway devices was statistically significant (*p* = 0.003) in favour of the LMA Supreme.

### Number of attempts required for successful placement

The insertion of the LMA Supreme and endotracheal intubation with the Macintosh laryngoscope had 100% first-attempt success, but successful intubation with the Airtraq required a second attempt in five participants and one participant had to re-insert the LT because the first insertion resulted in tracheal intubation (*p* = 0.007; Table 2).

**Table 2:** Number of attempts required for successful placement (*n* (%))

	1st attempt	2nd attempt	3rd attempt
LMA Supreme	26 (100)	0	0
Laryngeal tube	25 (96)	1 (4)	0
Macintosh	26 (100)	0	0
Airtraq	21 (81)	5 (19)	0

*SD* standard deviation, *CI* confidence interval, *LMA* laryngeal mask airway

### Degree of difficulty

The LMA Supreme was rated least difficult to insert with a mean score of 1.7/10 (CI [0.95] 1.2–2.1) followed by the LT with a mean score of 2.5/10 (CI [0.95] 1.8–3.2), endotracheal intubation using the Macintosh laryngoscope with a mean score of 3.7/10 (CI [0.95] 2.9–4.5) and the Airtraq with a mean score of 4.5/10 (CI [0.95] 3.7–5.3) (*p* < 0.001; Table 3). Data were pooled into extraglottic airway device and ETI groups, analysis of which showed a statistically significant difference between the two groups in terms of degree of difficulty (*p* < 0.001).

**Table 3:** Degree of difficulty

	Mean	Median	SD	95% CI
LMA Supreme	1.7	1.0	1.0	1.2–2.1
Laryngeal tube	2.5	2.0	1.7	1.8–3.2
Macintosh	3.7	3.5	2.0	2.9–4.5
Airtraq	4.5	4.0	2.0	3.7–5.3

*SD* standard deviation, *CI* confidence interval, *LMA* laryngeal mask airway

Scale 1–10: 1 = least difficult and 10 = most difficult

### Cormack-Lehane grades

**Fig. 2:**
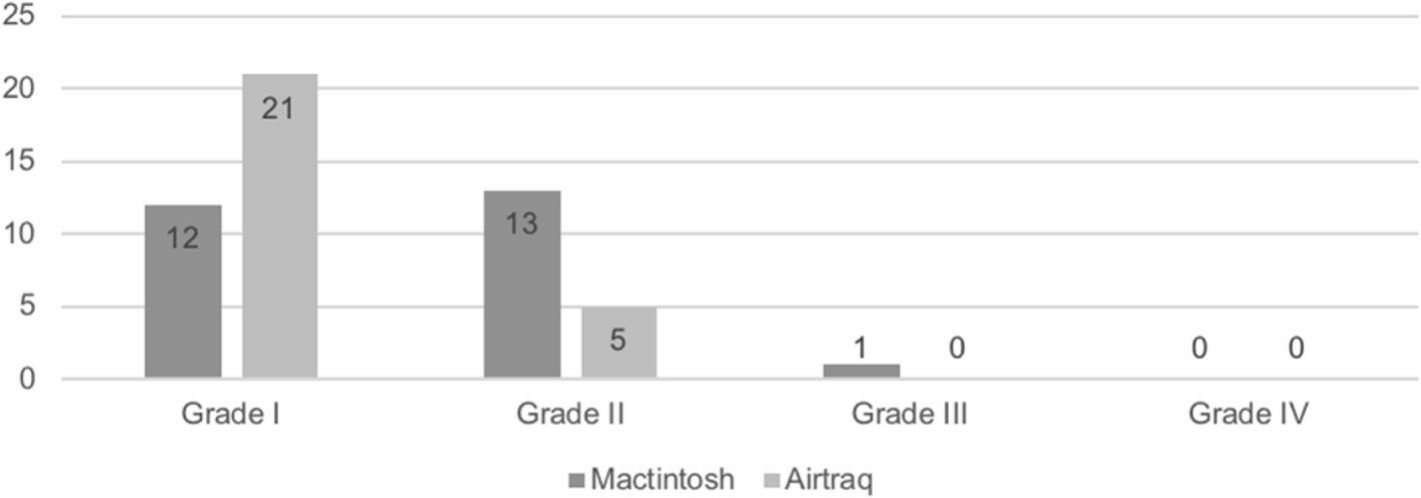
Number of Cormack-Lehane grade views using the Macintosh and Airtraq laryngoscopes

For laryngoscopy with the Macintosh and Airtraq laryngoscopes, CLG I was recorded 12 and 21 times (46.2% vs. 80.8%), respectively; CLG II 13 and 5 times (50.0% vs. 19.2%), respectively; and CLG III 1 and 0 times (3.8% vs. 0.0%), respectively (Fig. 2). CLG IV view was not recorded for any of the two devices.

### Multiple regression analysis

Multiple regression analysis was performed using an automatic forward stepwise model to determine if rated degree of difficulty was associated to the time to first ventilation variable as well as the number of attempts required for successful placement variable (Table 4). The results of the test indicated that there is a statistically significant association (adjusted *R*^*2*^ = 0.4311, *p* < 0.001) between rated degree of difficulty and time to first successful ventilation (*p* < 0.001; CI [0.95] 0.06 to 0.10) as well as between rated degree of difficulty and number of attempts required for successful placement (*p* < 0.001; CI [0.95] 1.32 to 3.90).

**Table 4:** Multiple regression analysis: rated degree of difficulty

Predictor variable	*b*	SE	*t*	95% CI	*p* value
Time to first successful ventilation	0.08	0.01	7.38	0.06–0.10	< 0.001
Attempts required for successful placement	2.61	0.65	4.02	1.32–3.90	< 0.001
Constant	− 1.92	0.73	− 2.64	− 3.37 to − 0.48	0.01

*N* of obs. = 104 airway placements. *F* = 40.03. *R*^*2*^ = 0.44. Adjusted *R*^*2*^ = 0.43

As part of the analysis, it was also investigated whether paramedic experience or the estimated number of entrapment-trauma airway management had any association with time to first ventilation, number of attempts required for successful placement, or rated degree of insertion difficulty. This was performed to identify whether either of these two attribute variables could possibly be confounders.

Using a multiple regression analysis, the association between paramedic experience in months and the above-mentioned variables was performed. The results indicated that there was no significant association (adjusted *R*^*2*^ = 0.0119, *p* = 0.24) overall or between any of the dependent variables with regard to paramedic experience. The same model was then repeated for estimated number of entrapment-trauma airway management. Similarly, no significant association was found (adjusted *R*^*2*^ = − 0.0132; *p* = 0.65) overall or between the individual variables. When this was repeated by device, for all devices in both models, no association was found.

### Clinician preference

Most paramedics chose the Macintosh laryngoscope as their preferred airway management device (10/26; 38%). This was followed very closely by the LMA Supreme (9/26; 35%). The Airtraq and LT were less preferred (5/26 (19%) and 2/26 (8%), respectively).

## Discussion

This study suggests that the LMA Supreme and the LT may be feasible alternatives to ETI in the entrapped patient after an MVC as evidenced by the advantages in time and ease of insertion. Many of the participating paramedics indicated the LMA Supreme as their preferred device for the entrapped patient requiring advanced airway interventions. When compared to the Airtraq, the extraglottic airway devices also produced superior first-attempt success rate. Our findings echo results of comparable manikin studies by Martin et al. [14], Steinmann et al. [18] and Wetsch et al. [29], who also report advantages of extraglottic airway devices over ETI in similar, simulated entrapment-trauma scenarios. Prehospital intubation can be, and very often is, significantly different to intubation performed in the controlled, in-hospital setting. This is especially true in cases where access to the patient is restricted, which may lead to increased incidence of failed intubation attempts [5]. Our study indicates that in the entrapped patient, ETI using the Macintosh or Airtraq laryngoscope takes significantly more time and is significantly more difficult compared to insertions of the extraglottic airway devices. It follows that the use of an extraglottic airway device as the primary advanced airway in entrapment-trauma should be considered. The concept of rapid sequence airway (RSA), an airway management technique in which the preparation and pharmacology of rapid sequence intubation (RSI) is paired with intentional placement of an extraglottic airway device without prior attempt at endotracheal intubation, was previously explored by Southard, Braude and colleagues [22, 30]. Endotracheal intubation is the gold standard for securing the airway with unmatched features. Not only does the endotracheal tube provide unsurpassed airway protection from aspiration, but also allows for ventilation at high airway pressures, offers a means to perform tracheal suctioning and potentially a route for drug administration. Extraglottic airway devices offer inferior airway protection and ventilation pressure capacity compared to the endotracheal tube. However, newer extraglottic airway devices, such as the ones examined in this study, form an improved oesophageal seal compared to the classic LMA without gastric drainage [31]. Extraglottic airway devices should not lead to the avoidance of endotracheal intubation or de-emphasize the importance of appropriately trained prehospital clinicians having and maintaining the competence to insert an endotracheal tube should the patient’s needs demand this [32]. Nevertheless, extraglottic devices may offer an expedient alternative when endotracheal intubation is anticipated to be difficult. Conversion to a definitive airway such as an ETT could be performed after extrication in the prehospital or hospital setting for such patients.

The incidence of unsatisfactory view of the larynx during prehospital laryngoscopy is high when compared with intubations performed in the operating theatre. Difficult laryngoscopy and position of the patient have previously been reported to be the leading cause of difficult airway management in the prehospital setting [33]. The response to an anticipated difficult intubation situation requires forethought and planning [26]. The utilization of indirect laryngoscopy as a best first-attempt method has been studies extensively [9, 10]. Without the need for axes alignment, indirect laryngoscopy can provide a high-grade view of the glottic opening and do so with significantly less cervical spine movement [34]. In our study, the Airtraq laryngoscope enabled the CLG I view significantly more often compared to the Macintosh laryngoscope. CLG II and III views were experienced less frequently with the Airtraq laryngoscope. However, the Airtraq had a significantly lower first-attempt success rate when compared to the Macintosh laryngoscope. Our findings that the Airtraq laryngoscope used by paramedics in the simulated entrapped patient provides superior laryngoscopy but inferior first-attempt intubation success in comparison to the Macintosh laryngoscope are in line with findings from Steinmann et al. [18]. Although the use of the Airtraq resulted in the lowest first-attempt success rate, the 100% success rate with no more than two attempts suggests a steep learning curve.

Despite the advantages of the extraglottic airway devices in the simulated scenario, most participating paramedics preferred endotracheal intubation using the Macintosh laryngoscope. Based on information gathered from the participating paramedics about what airway devices they use in their clinical practice, it may be reasonable to suspect that their preference is due to being more familiar with endotracheal intubation using the Macintosh laryngoscope or due to concerns about airway protection and ventilation limitations with extraglottic airway devices. Similarly, the preference of the LMA Supreme over the LT is likely to be due to familiarity with supraglottic laryngeal masks rather than retroglottic devices.

There are several limitations that must be taken into account when considering the findings of this research. The procedures were performed on a manikin in a simulated entrapment scenario in daylight. The awareness of participants being observed inherently introduced potential of the Hawthorne effect. Furthermore, simulation cannot duplicate a real incident exactly. The mock patient had no airway reflexes or movement and was apnoeic. No vomiting, oropharyngeal haemorrhage or airway distortion was present. The car was not damaged or deformed and was positioned on its wheels. The environment around the vehicle was not austere. Nevertheless, use of the manikin, vehicle and setting allowed standardization of the patient and airway characteristics, as well as the surrounding across all participants, and thus acted as constants rather than variables.

A convenient/volunteer sampling strategy was used. The participants volunteering to take part in this study may intrinsically have different characteristics from the general paramedic population. Thus, the non-probability sampling that was used in this study leads to limitations in generalizing the research findings and introduced the potential for volunteer bias. In particular, it needs to be highlighted that this limitation inhibits the generalization of the finding that neither the months of operational experience nor the estimated level of exposure to airway management in entrapped patients had a significant association with performance in terms of success rates, time to first ventilation or rated degree of difficulty. A formal sample size was not calculated. Therefore, the study remains in all likelihood substantially underpowered and subject to type 2 error. However, the results are hypothesis-generating and could be used to inform future, appropriately powered studies.

## Conclusion

The investigated extraglottic airway devices can be considered as beneficial primary advanced airway devices to be used by paramedics in the entrapped patient after a motor vehicle collision. Both, the LMA Supreme and the LT could be placed swiftly, easily and with high success. Face-to-face endotracheal intubation with the Macintosh laryngoscope remains an important definitive airway that was shown to be performed competently by participating paramedics. The Airtraq can be used for face-to-face ETI and enables improved laryngoscopy.

The results of this manikin study call for further evaluation of the LMA Supreme, the LT or other modern extraglottic airway devices in prehospital trauma care. Primarily, this entails the assessment of the risk of aspiration and the ability to facilitate ventilation at increased airway pressures. Further investigation into the use of the Airtraq is also warranted, especially as an alternative intubation device in the patients with anticipated difficult direct laryngoscopy.

## Data Availability

All data generated or analysed during this study are included in this published article.

## Abbreviations

ALS: Advanced life support
CLG: Cormack-Lehane grade
ETI: Endotracheal intubation
LMA: Laryngeal mask airway
LT: Laryngeal tube
MVC: Motor vehicle collision
